# The Effect of Capsaicin on Salivary Gland Dysfunction

**DOI:** 10.3390/molecules21070835

**Published:** 2016-06-25

**Authors:** Yong-Hwan Shin, Jin Man Kim, Kyungpyo Park

**Affiliations:** Department of Physiology, School of Dentistry, Seoul National University and Dental Research Institute, Seoul 110-749, Korea; shinyh@snu.ac.kr (Y.-H.S.); kim-jm@snu.ac.kr (J.M.K.)

**Keywords:** Capsaicin, Salivary glands, TRPV1, NF-κB

## Abstract

Capsaicin (trans-8-methyl-*N*-vanilyl-6-nonenamide) is a unique alkaloid isolated from hot chili peppers of the capsicum family. Capsaicin is an agonist of transient receptor potential vanilloid subtype 1 (TRPV1), which is expressed in nociceptive sensory neurons and a range of secretory epithelia, including salivary glands. Capsaicin has analgesic and anti-inflammatory properties in sensory neurons. Recently, increasing evidence has indicated that capsaicin also affects saliva secretion and inflammation in salivary glands. Applying capsaicin increases salivary secretion in human and animal models. Capsaicin appears to increase salivation mainly by modulating the paracellular pathway in salivary glands. Capsaicin activates TRPV1, which modulates the permeability of tight junctions (TJ) by regulating the expression and function of putative intercellular adhesion molecules in an ERK (extracelluar signal-regulated kinase) -dependent manner. Capsaicin also improved dysfunction in transplanted salivary glands. Aside from the secretory effects of capsaicin, it has anti-inflammatory effects in salivary glands. The anti-inflammatory effect of capsaicin is, however, not mediated by TRPV1, but by inhibition of the NF-κB pathway. In conclusion, capsaicin might be a potential drug for alleviating dry mouth symptoms and inflammation of salivary glands.

## 1. Introduction

Salivary glands are exocrine glands, and saliva plays a critical role in maintaining oral health. Xerostomia—or dry mouth—is common in the elderly, but the etiology of xerostomia has remained elusive. Aging, radiation therapy for head and neck cancer, and drugs such as antidepressants may induce xerostomia. Decreased salivary flow results in deteriorating oral health, including rampant caries, burning mouth syndrome, and candidiasis. Xerostomia also accompanies autoimmune diseases—for example, primary Sjögren’s Syndrome (pSS) [1,2,3,4], which also affects another exocrine organ, the lacrimal glands [5,6]—and significant changes of cytokines and nitric oxide [7].

Capsaicin (trans-8-methyl-*N*-vanilyl-6-nonenamide) is a colorless alkaloid (capsaicinoid) found in various capsicum chilies that gives “heat” to peppers widely consumed for hot flavor or spice. Capsaicin induces inflammation of the oral cavity that can lead to stomatitis and orofacial pain. Capsaicin has also been shown to impair sensory nerve endings. However, continually applying capsaicin deteriorates the cutaneous autonomic nerve fibers, decreasing pain sensation [8]. The pharmacological actions of capsaicin have been broadly explored in sensory neurons, and now capsaicin is widely used as a drug to alleviate chronic pain [9,10]. Topically applying capsaicin to the skin has an analgesic effect; capsaicin creams and lotions are broadly prescribed to treat arthritis, neuralgia, pruritus and pain due to their antioxidant and anti-inflammatory properties. Recently, capsaicin has also been shown to enhance salivary secretion and have anti-inflammatory effects in salivary glands or via the trigeminal-parasympathetic pathway [11,12,13,14], but its mechanism of action has not been rigorously studied.

Transient receptor potential (TRP) channels are nonselective cation channels that participate in various cellular functions, including sensory transduction, mechanosensation, and thermosensation, and capsaicin is a well-known agonist for transient receptor potential vanilloid subtype 1 (TRPV1). TRPV1 is a member of the TRP family that can also be activated by acidic conditions and heat [15]. TRPV1 consists of 838 amino acids and it was identified and cloned in human and rodent [16]. Although TRPV1 is mainly expressed in specialized sensory neurons, recent reports showed that TRPV1 is also expressed in various non-neuronal cells, including bronchial and bladder epithelial cells, dental pulp fibroblasts, and salivary glands [17,18,19,20,21,22]. Applying capsaicin promoted saliva secretion in rabbit, rat, and human submandibular glands (SMG) [17,18,22].

In this paper, we provide a comprehensive review of the properties and mechanism of action of capsaicin, along with a review of its potential as a drug for salivary gland dysfunction and inflammation.

## 2. Modulation of Salivary Secretion by Capsaicin

Primary saliva is produced by end piece cells in salivary gland acinar cells, and is finally secreted into the mouth through ducts. Secretion of saliva is induced by stimulating muscarinic cholinergic receptors or α-adrenoceptors in the basal membrane of the acinar cells [23]. Salivary secretion is also controlled by diverse peptides through linked receptors (such as TRPV1) in sensory neurons or directly in the salivary glands under diverse physiological conditions. TRPV1 is mainly expressed in trigeminal ganglion neurons and small-diameter dorsal root ganglia, where it plays a critical role in recognizing noxious painful stimuli. Recently, TRPV1 expression was reported in the secretory epithelia; for example, ductal and acinar cells in human and rabbit submandibular glands. However, the role of TRPV1 in secretory epithelia has remained elusive.

Increasing evidence indicates that capsaicin—a TRPV1 agonist—enhances salivary secretion. Capsaicin increased salivary secretion in humans and in a rabbit model [18,24,25,26]. Capsaicin appears to increase salivary secretion directly by affecting paracellular permeability of the tight junction (TJ). Increased permeability of the TJ induced by capsaicin has also been reported in human intestinal cells [27]. Activating TRPV1 modulates the expression and function of intercellular adhesion molecules in TJ. Reduced TJ expression and its destruction in transplanted submandibular glands are improved by applying capsaicin [17,28]. While capsaicin can indirectly increase salivary secretion, topically applying capsaicin to the skin around the mouth increased salivary secretion from the submandibular and sublingual glands in humans [25]. Capsaicin considerably increased resting salivary secretion in healthy volunteers compared to subjects with dry mouth in a hyposalivation group [26]. Contrary to the effects of capsaicin on salivary glands, TRPV1 knockout mice did not have a significant difference in salivary flow induced by capsaicin when compared to wild-type mice [11]. These results suggest that, although capsaicin increases salivary flow in humans and some animal models, its effects depend on the species.

## 3. Mechanism of Capsaicin Action in Salivary Secretion

**Figure 1:**
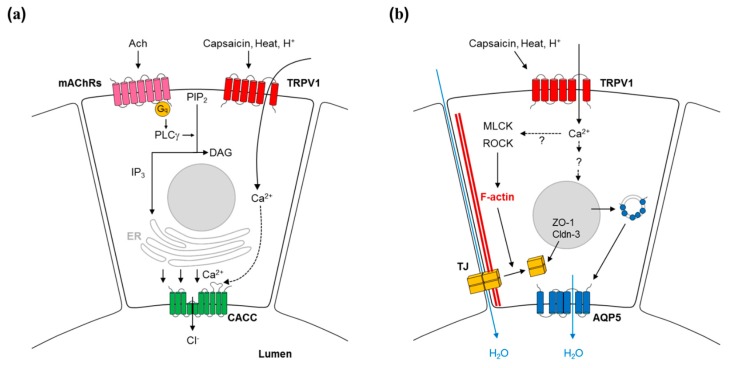
Capsaicin mediates saliva secretion via transient receptor potential vanilloid subtype 1 (TRPV1). (**a**) Calcium-activated chloride channel (CACC)-induced saliva secretion. Activation of Muscarinic acetylcholine receptors (mAChRs) via acetylcholine (Ach) mediates G-protein coupled signaling downstream, including the phospholipase Cg (PLCg) pathway. The generated inositol 1,4,5-triphosphate (IP_3_) by PLCg induces Ca^2+^ mobilization from ER storage. Capsaicin increased [Ca^2+^]_i_ level by increasing the cation conductance of TRPV1. The increased [Ca^2+^]_i_ promotes the saliva secretory process by activating CACC, leading the efflux of chloride ions toward ductal space, and the subsequent generation of an electrochemical gradient beyond the apical border of acinar cells. (**b**) The role of TRPV1 in trans- and paraceulluar routes. TRPV1 activation enhances paracellular permeability by regulating the spatial distribution of F-actin linked to tight junction (TJ). The expression level of TJ proteins—including zonula occludin (ZO)-1 and Claudin (Cldn)-3—is also increased by TRPV1 activation. In the transcellular route, capsaicin treatment enhances the aquaporin 5 (AQP5) trafficking to the plasma membrane and its mRNA expression level. However, the accurate mechanism underlying AQP5 trafficking is still unclear. Broken lines are used for tentative interactions. For details, see text. PIP2: Phosphatidylinositol 4,5-bisphosphate; DAG: diacylglycerol; MLCK: myosin light chain kinase; ROCK: RhoA-Rho-associated protein kinase.

Muscarinic acetylcholine receptors (mAChRs) are a well-established upstream mediator of salivary secretion [29]. Various studies have reported the distribution of mAChRs in salivary glands. M1 and M3 receptors are expressed in the sublingual and submandibular glands, and M3 receptor expression was predominant in the parotid gland [30]. Moreover, knockout mouse studies have verified that mAChRs mediate cholinergic stimulation of salivary flow [31,32]. Activating mAChRs mediates signaling downstream of G-protein coupled receptors, including the phospholipase g (PLCg) pathway. The inositol-3-phosphates (IP_3_) generated by PLCg induce [Ca^2+^]_i_ to mobilize from ER storage through IP_3_ receptors on the ER membrane. The increased [Ca^2+^]_i_ level initiates saliva secretion by activating calcium-activated chloride channels (CACC) [23,33]. CACC activation is the rate-limiting step for fluid secretion in various exocrine tissues [34]. CACCs are locally expressed on the apical membrane of acinar cells, and CACC activation evokes the efflux of chloride ions toward the ductal space, generating an electrochemical gradient across the apical membrane of acinar cells [35]. The spatial difference in ionic concentration triggers osmotic pressure that promotes water transport to the intercalated duct. In salivary glands, TMEM-16A (a CACC) was found to be an essential component in saliva secretion induced by a muscarinic agonist [34] (Figure 1a).

The TRPV1 channel plays an important role in capsaicin-induced saliva secretion, which is independent of PLCg-IP_3_ signaling. The TRP superfamily encodes TRP proteins, which act as multimodal sensing channels for a variety of stimuli—such as pain, acidosis, temperature, and osmolality from outside and inside the cell. Such chemical or physical stimulation enables TRP channels to increase the [Ca^2+^]_i_ level by activating cation conductance [15]. TRPV1 plays a main role in inflammatory hyperalgesia and thermal nociception [36,37]. TRPV1 is a ligand-gated nonselective cation channel that is permeable to calcium and sodium ions. Capsaicin, noxious heat, protons and endogenous lipid compounds regulate TRPV1 activity directly or indirectly. TRPV1 is broadly distributed in various tissues, such as the bowel, kidney, bladder, brain and salivary glands [22,38]. Capsaicin reportedly increased [Ca^2+^]_i_ levels in cells isolated from the human submandibular gland (SMG). This effect was abolished by capsazepine (TRPV1 antagonist) treatment. These results demonstrate that capsaicin can evoke Ca^2+^ influx via TRPV1 in human SMGs [18]. Capsazepine also blocked salivation after capsaicin treatment in isolated rabbit SMGs, indicating that TRPV1 mediates capsaicin-induced saliva secretion [22]. In addition to the role of TRPV1 in saliva secretion, the interaction between TRPV and CACC has also been explored. TRPV4 agonist increased the chloride current mediated by TMEM16A (transmembrane member), resulting in water efflux from choroid plexus epithelial cells [39]. Moreover, TRPV1-TMEM16A interaction enhanced neuronal depolarization in mouse dorsal root ganglion neurons [40]. Immunoprecipitation data in both studies revealed direct physical binding between TRPV and CACC, strengthening the functional relationship between the two channels. In the salivary gland, whether the TRPV-CACC functional complex is involved in fluid secretion is unclear. Resolving this question will be important in dissecting the mechanism triggering saliva secretion. The gustatory–salivary reflex is well known. Chemical sensation, or taste, is a strong agonist of salivation. Our group found that capsaicin inhibits outward-rectifying K^+^ channels in taste receptor cells (TRCs) isolated from circumvallate papillae in rat. Inhibiting the K^+^ channel may evoke TRC excitation, activating the solitary nucleus tract (SNT) in the brain. Thus, activating the TRC via TRPV1 may activate the salivatory nucleus adjacent to the SNT. These results suggest that stimulating TRPV1 in TRC may induce salivary secretion through the gustatory–salivary reflex [11].

In the late phase of saliva secretion, water including saliva components moves across the epithelial barrier through the apical membrane and intercellular junction of acinar cells—known as the transcellular and paracellular routes, respectively [41]. Various studies have reported that capsaicin dynamically modulates the permeability of both routes via TRPV1. In the paracellular route, TJ proteins organize a primary barrier to the diffusion of saliva components, and these proteins are a major modulator of TRPV1-mediated paracellular transport. TRPV1 activation increases the paracellular permeability by regulating the spatial distribution of the cytoskeleton linked to the TJ as well as the expression of TJ components, such as zonula occludens (ZO) and claudin [17]. Especially, the intracellular location of occludin is tightly regulated by F-actin reorganization due to the capsaicin-TRPV1-myosin light chain kinase (MLCK) pathway [42]. ZO-1 and -2 were redistributed after TRPV1 activation, but the RhoA-Rho-associated protein kinase (ROCK) pathway was also involved [43]. Such delicate regulation of TJ proteins by cytoskeletal organization has a crucial role in regulating the permeability of paracellular space and determining the flow rate of saliva (Figure 1b).

The transcellular route has also been suggested to explain capsaicin-mediated saliva secretion. An integral membrane water channel—aquaporin 5 (AQP5)—plays a critical role in fluid transport through the transcellular pathway [44]. Capsaicin treatment enhanced AQP5 translocation to the plasma membrane and AQP5 mRNA expression in salivary gland epithelial cell lines and primary culture cells [18,25] (Figure 1b). Despite these results, the evidence does not support capsaicin increasing permeability via the transcellular pathway. Secretion through the transcellular pathway requires ion channel activation to move ions. However, capsaicin has not yet been reported to activate ion channels (for example, CACC) in salivary glands. TRPV1 stimulation has rarely been reported to increase Ca^2+^. Capsaicin hardly increased Ca^2+^ in mice, although acidity and high temperature did [11].

## 4. Modulation of Inflammation by Capsaicin

Capsaicin has an anti-inflammatory effect in sensory neurons [45,46]. TRPV1 has a central role in cell signaling of inflammatory responses and peripheral tissue injury [47]. However, repeatedly applying capsaicin has an anti-inflammatory effect [48].

The anti-inflammatory effects of capsaicin can also be mediated without TRPV1 activation. To date, capsaicin has been shown to participate in immunological responses independent of TRPV1 in specific pathological situations, and this response is mainly related to nuclear factor kappa B (NF-κB) signaling. Capsaicin reportedly inhibits inflammatory responses induced by NF-κB activation. Capsaicin treatment efficiently blocked cytokine production induced by proinflammatory stimuli or direct NF-kB activation in immune cells [49,50,51]. Moreover, capsazepine did not block inhibition of pro-inflammatory cytokines released by capsaicin, suggesting that a signaling pathway other than TRPV1 is involved in the capsaicin-mediated anti-inflammatory process [13].

The anti-inflammatory action of capsaicin in salivary glands appears not to be mediated by TRPV1. The anti-inflammatory properties of capsaicin have been reported in vitro by using salivary gland epithelial cells (SGEC) [13]. Capsaicin decreased the expression of pro-inflammatory cytokines such as TNFα and IL-6, induced by polyinosinic-polycytidylic acid (poly (I:C)) or lipopolysaccharide (LPS), but capsazepine did not inhibit the anti-inflammatory activity of capsaicin. In addition, the anti-inflammatory activity of capsaicin was similar in TRPV1 KO mice. Capsaicin inhibited IκB-α (nuclear factor of kappa light polypeptide gene enhancer in B-cells inhibitor, alpha) phosphorylation and degradation induced by poly (I:C) or LPS, which act through a TRPV1-independent signaling mechanism. Capsazepine also has hardly any effect on IκB-α phosphorylation. NF-κB also plays a critical role in the pathogenesis of salivary glands, and increased NF-κB activation has been reported in salivary gland diseases [52,53,54,55,56]. Therefore, the anti-inflammatory action of capsaicin in SGEC is mediated by restraining the IκB-α/NF-κB pathway rather than TRPV1-dependent signaling [13].

The anti-inflammatory effect of capsaicin was not inhibited by capsazepine in murine peritoneal macrophages [50]. Instead, reports revealed that capsaicin inhibits IκB phosphorylation and subsequent degradation through immunoblot data [13,49,50,51]. These results suggest the existence of a non-canonical capsaicin pathway that directly regulates IκB phosphorylation/degradation without activating TRPV1. Nuclear factor kappa B (NF-κB) is a family of DNA-binding proteins and a protein complex that controls the transcription of diverse genes related to immune responses. Abnormal NF-κB activity elicits diverse inflammatory cytokines and chemokines in pathological conditions, such as inflammatory disorders, autoimmune diseases, and cancer [57]. NF-κB exists in the cytosol as either homo- or heterodimers-bound inhibitor of κB (IκB). The hallmark of NF-κB activation is IκBα degradation by pro-inflammatory signals, which releases NF-κB dimers in a free state capable of nuclear translocation. IκB degradation is initiated by phosphorylation by the IκB kinase (IKK) complex, leading to polyubiquitination and IκB degradation by the proteasome [58,59].

## 5. Pharmacokinetics and Therapeutic Potential of Capsaicin

Capsaicin is currently available as patches or cream containing 0.025%–1% capsaicin, and frequently is used for pain relief. Capsaicin can be administered by various ways, including oral and topical administration. The oral administration of capsaicin in humans is very rare, but a recent study provided pharmacokinetic analysis of orally ingested 5 g capsicum in human. The study showed that capsaicin was detected in plasma after 10 min of oral administration, maximum concentration of capsaicin was reached at 2.5 ng/mL, which then dropped to zero after 90 min [60].

Human skin is known to be highly permeable to topically administered capsaicin, and recent reports showed that 12 human subjects with topically administered 3% capsaicin solubilized in various solvents such as mineral oil, propylene glycol in 20% alcohol, and 70% isopropyl alcohol. When capsaicin solubilized in 70% isopropyl alcohol was applied topically on human skin, it got quickly absorbed and rapidly reached maximum concentration of 16.1 μg, and the half-life time of capsaicin was 24 h [61].

A highly-concentrated capsaicin patch (NGX-4010) containing 179 mg of capsaicin has been clinically used for the treatment of neuropathic pain patients. A clinical study of the capsaicin patch reported that topical application of NGX-4010 provided quick and continuous pain relief to human patients with postherpetic neuralgia (PHN). Sixty minutes after the topical application of NGX-4010, plasma concentration of capsaicin was reached at approximately 1.38 ng/mL [62]. Another study of clinical trials reported that a low-concentration capsaicin patch was not capable of reducing pain in patients with neuropathy. Safety and availability of low concentration capsaicin patches are more tolerated, but it offered no significant pain relief at all [63]. These reports suggest that the high concentration capsaicin patch is more capable of relieving pain than the low concentration capsaicin patch.

Capsaicin′s pharmacological effect on salivary glands was also examined in several studies. They reported that topical application of 1.4 μg capsaicin on the skin around the mouth was enough to induce a significant increase in salivary secretion from human salivary glands. However, a capsaicin dose over 2.0 μg caused skin irritation [25,26]. Taken together, although there is a risk of skin irritation, the topical administration of capsaicin provides simple and efficient ways to treat xerostomia and neuropathy in the clinical field.

Capsaicin has been used as a therapeutic agent, and new evidence supports the use of capsaicin in a wide variety of clinical circumstances. Capsaicin has potential antioxidant, anti-proliferative, anti-inflammatory, and anti-cancer activities [12,64]. The anti-inflammatory and antioxidant properties of capsaicin can be used to mitigate tissue damages after transplantation, as other anti-inflammatory drugs prevent graft resorption by reducing inflammation [65]. Recently, capsaicin was reported to attenuate lung ischemia–reperfusion (IR) injury, which is major complication after lung transplantation [66]. In a rabbit autotransplantation model, capsaicin treatment promoted functional recovery of transplanted salivary glands, implying its potential effectiveness as a transplantation medication [28]. Capsaicin has also been used topically to treat neuropathic pain; however, the side effects—such as skin irritation—remain to be solved. Capsaicin and its analogues have been used in creams and high concentration patches (such as NCX-4010) to treat chronic pain syndromes such as postherpetic neuralgia, osteoarthritis, musculoskeletal pain, and rheumatoid arthritis, but these applications have not been thoroughly investigated [67,68]. Therefore, the analgesic properties of capsaicin need to be investigated in diverse pain conditions.

Capsaicin elevated cytoplasmic free [Ca^2+^], ERK phosphorylation, AQP5 trafficking, and salivary secretion in salivary glands through TRPV1 [13,18]. Thus, TRPV1 activation might be a novel signaling mechanism regulating salivary gland function and may lead to a new therapeutic tactic to treat salivary gland dysfunction and recover natural salivary secretion. Although capsaicin might be a potential drug to treat dry mouth symptoms and inflammation generated by pro-inflammatory cytokines in salivary glands, whether capsaicin affects chronic inflammatory disorders such as Sjögren’s syndrome remains uncertain. An understanding of the exact mechanism of action of capsaicin in various cells—including SGEC—will allow for the development of more efficient and acceptable therapies.

## 6. Conclusions

Although capsaicin may be an important molecule in medicine, the current clinical applications of capsaicin are limited to pain management, and this limitation can be attributed to its associated potential toxicity and lack of specificity. Moreover, the application of capsaicin to other indications has been restricted because of the unclear mechanism of action of capsaicin in different physiological systems [8]. Further studies are essential to explore the interaction of capsaicin with TRPV1, which might uncover other pharmacological properties and other potential advantages of capsaicin. Future research must clarify our understanding of the signaling pathways related to capsaicin activity. Many studies are modifying the capsaicin molecule to overcome the adverse properties, mostly its pungency and chili taste. Non-pungent analogs of capsaicin molecules have been considered and are encouraging molecules with many clinical applications, but the lack of clinical trials prevents the extensive pharmacological use of capsaicin.
